# BET Inhibition Upregulates SIRT1 and Alleviates Inflammatory Responses

**DOI:** 10.1002/cbic.201500272

**Published:** 2015-08-13

**Authors:** Tarja Kokkola, Tiina Suuronen, Maija Pesonen, Panagis Filippakopoulos, Antero Salminen, Elina M Jarho, Maija Lahtela-Kakkonen

**Affiliations:** [a] School of Pharmacy, University of Eastern FinlandYliopistonranta 1C, 70211, Kuopio, Finland E-mail: tarja.kokkola@uef.fi; [b] Institute of Clinical Medicine, University of Eastern FinlandYliopistonranta 1C, 70211, Kuopio, Finland; [c] Structural Genomics Consortium, University of OxfordRoosevelt Drive, Oxford, OX3 7DQ, UK

**Keywords:** acetylation, bromodomain and extra terminal domain protein, cancer, epigenetics, gene expression, SIRT1

## Abstract

Control of histone acetylation is a part of the epigenetic mechanism that regulates gene expression and chromatin architecture. The members of the bromodomain and extra terminal domain (BET) protein family are a group of epigenetic readers that recognize histone acetylation, whereas histone deacetyl- ases such as sirtuin 1 (SIRT1) function as epigenetic erasers. We observed that BET inhibition by the specific inhibitor JQ1 upregulated SIRT1 expression and activated SIRT1. Moreover, we observed that BET inhibition functionally reversed the pro-inflammatory effect of SIRT1 inhibition in a cellular lung disease model. SIRT1 activation is desirable in many age-related, metabolic and inflammatory diseases; our results suggest that BET protein inhibition would be beneficial in treatment of those conditions. Most importantly, our findings demonstrate a novel mechanism of SIRT1 activation by inhibition of the BET proteins.

BET proteins (BRDT and BRD2–4) activate transcription, whereas sirtuins have the opposite effect, gene silencing.[1] BET proteins are important for cell-cycle control and they have been linked to the development of a number of extremely aggressive tumors.[2] Sirtuins have been associated with the elongation of life-span, and the activation of sirtuin 1 (SIRT1) has caught particular interest world-wide.[3,4] Both BET proteins and SIRT1 have been connected to several age-related, inflammatory and metabolic diseases, thus making these epigenetic regulators interesting targets for drug development. During the past years, we and others have developed various SIRT1 inhibitors, and several inhibitors for BET proteins have been reported.[1,5–8] As the functions of both BET proteins and SIRT1 depend on the histone acetylation status, we explored whether inhibition of the BET proteins by a selective inhibitor and gene silencing could affect human SIRT1. After the finding of SIRT1 upregulation and activation, we analyzed the biological effects of this pathway in A549 cells, a lung disease cell line where SIRT1 is known to have beneficial anti-inflammatory effects.[9]

BET inhibition by JQ1 (**1**)
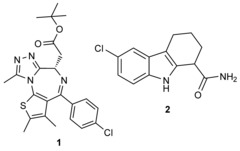
was used to explore the interplay between BET proteins and human SIRT1 in different cell types. JQ1 treatment for 24 h evoked a strong dose-dependent increase in SIRT1 expression in mouse N9 microglial cells (Figure 1 A), and upregulated SIRT1 expression in cancerous (A549, MCF-7) and non-cancerous (HEK293) human cells (Figure 1 B).

**Figure 1:**
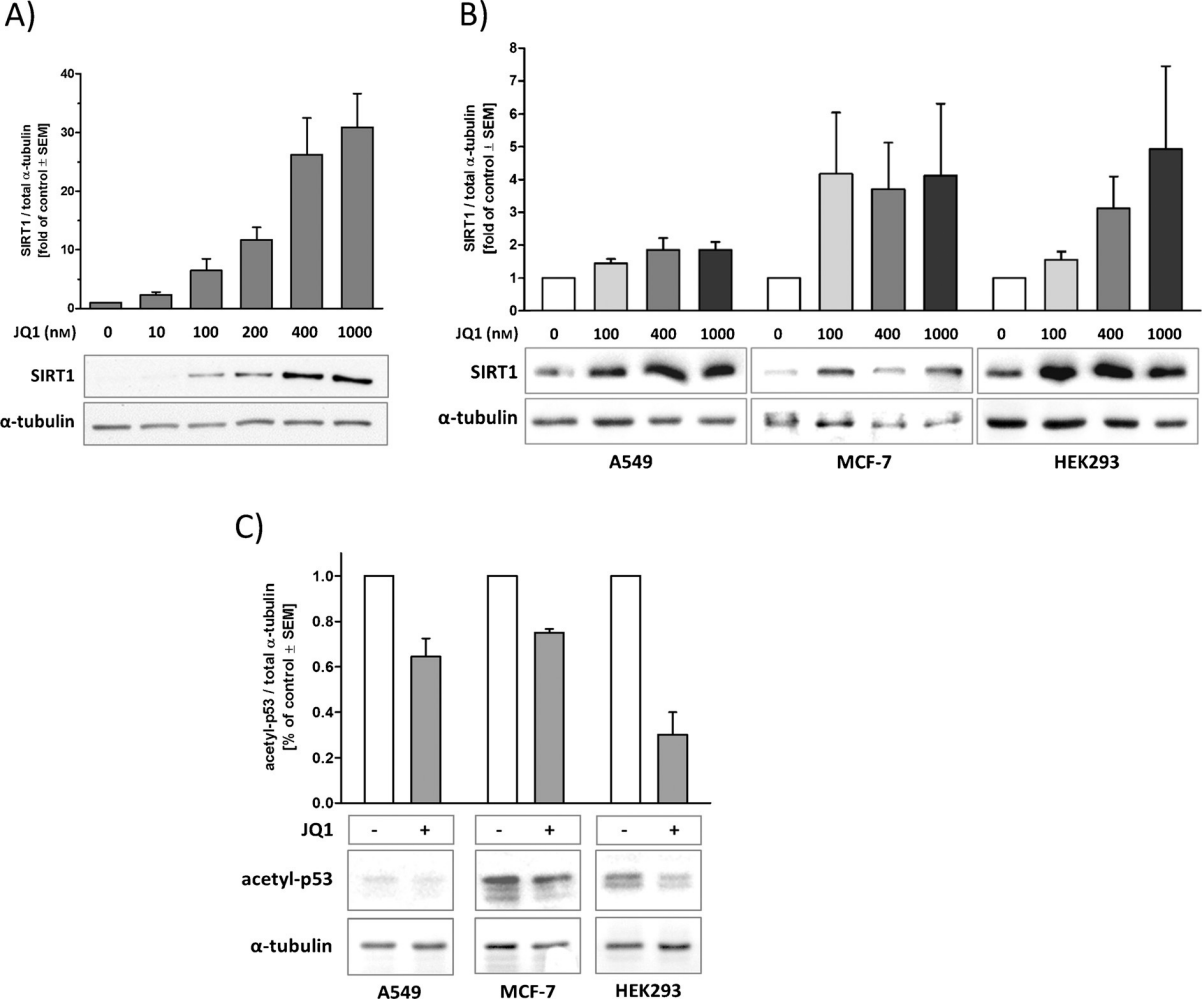
JQ1 treatment upregulates and activates SIRT1. JQ1 treatment for 24 h upregulates SIRT1 expression in A) mouse N9 microglia and B) A549, MCF-7 and HEK293 human cells. Cell lysates were analyzed by western blotting using SIRT1 and α-tubulin antibodies. C) JQ1 treatment enhances p53 deacetylation. A549, MCF-7 and HEK293 cells were treated for 24 h with JQ1 (400 nm), followed by a 5 h stimulation with etoposide (100 μm). Cell lysates were analyzed by western blotting with acetylated p53 and α-tubulin antibodies. Western blots are representative of at least three independent experiments.

Next we studied whether the deacetylase activity of SIRT1 increases with increased expression. The acetylation level of p53, a SIRT1 substrate, was analyzed by western blotting of human cell lysates. JQ1 enhanced p53 deacetylation (indicative of SIRT1 activation) in all tested cell lines (Figure 1 C). JQ1 did not affect the activity of SIRT1 in enzymatic activity assays (SIRT1 activity with 400 nm JQ1 was 99.3 %±2.1 % of control). This indicates that JQ1 is not a direct activator of SIRT1 *in vitro*. Visual inspection at 24 h showed that JQ1 treatments did not elicit any changes in cell morphology, and cell number was not affected by treatment, as determined by protein content in the wells (data not shown). In addition, flow cytometry analysis of cellular DNA content revealed that treatment with 400 nm JQ1 for 24 h did not modify the cell cycle or induce apoptosis in A549 cells (Figure S1 in the Supporting Information).

In order to induce inflammation, A549 (adenocarcinomic alveolar epithelial cells) were treated with lipopolysaccharide (LPS) for 24 h, and IL-8 secretion was quantified as an indicator of the inflammatory response. BET inhibition by JQ1 treatment prevented LPS-induced inflammation, whereas SIRT1 inhibition by the specific inhibitor EX527 (**2**) enhanced the inflammatory response. JQ1 treatment was able to reverse the inflammation-enhancing effect of SIRT1 inhibition (Figure 2 A). Furthermore, LPS treatment increased ROS production whereas treatments with EX527 or JQ1 had no significant effect on ROS generation (Figure 2 B). Gene silencing by siRNA transfections was used in order to assess the contributions of BRD2 and BRD4 in the JQ1-evoked responses. Four commercial siRNAs were tested for silencing efficiency of BRD2 and BRD4 genes, and the most efficient siRNAs were chosen for further experiments (see Figure S2). Silencing of BRD2 abolished (and silencing of BRD4 diminished) the inflammation-enhancing effect of SIRT1 inhibition without affecting basal or LPS-stimulated IL-8 secretion (Figure 3 A). BRD2 silencing was also found to reduce the stimulatory effect of LPS on ROS generation (Figure 3 B); BRD4 silencing did not have a significant effect on ROS levels (Figure 3 B).

**Figure 2:**
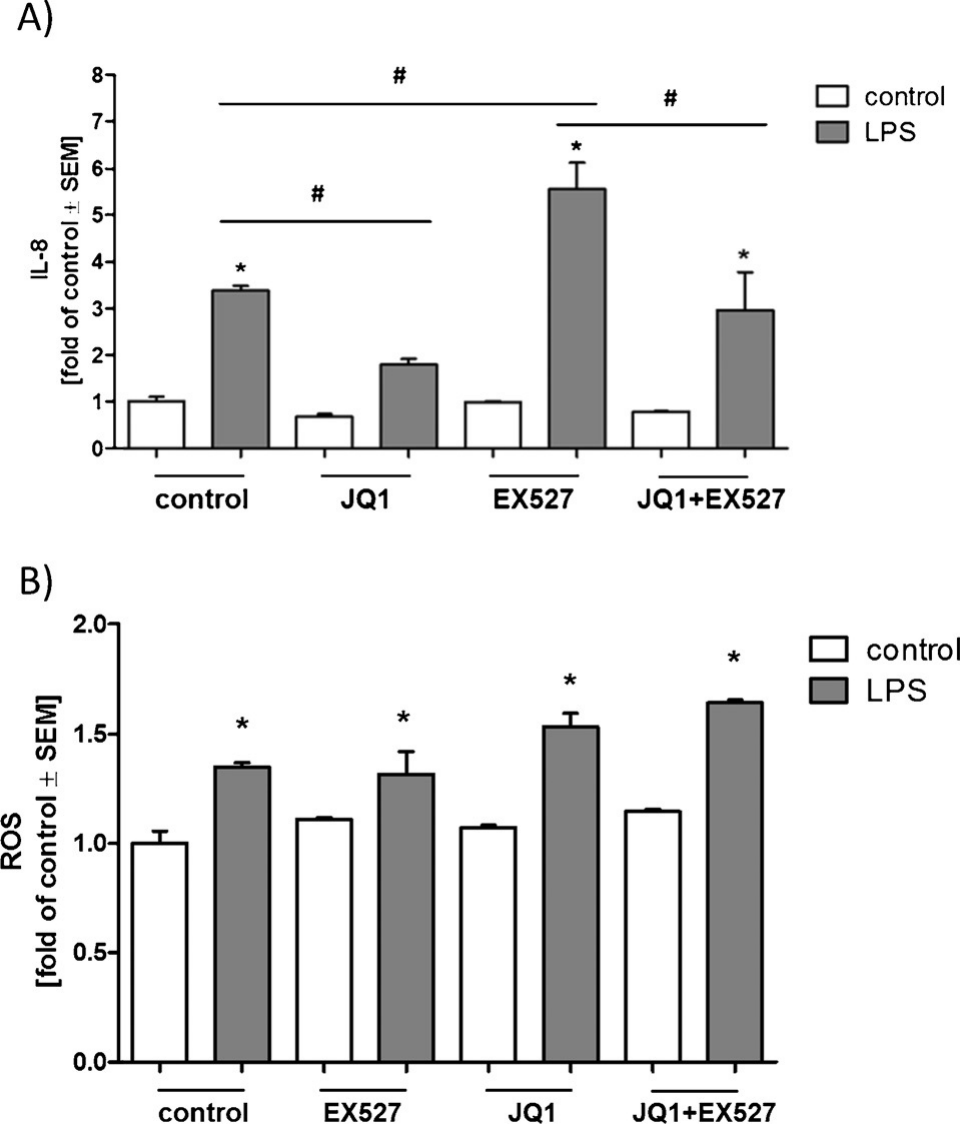
Effects of BET and SIRT1 inhibition on LPS-induced inflammatory response and ROS generation. A) JQ1 treatment diminished LPS-induced IL-8 secretion and reversed the effect of SIRT1 inhibition. A549 cells were treated with EX527 (5 μm), JQ1 (400 nm) and LPS (10 μg mL^−1^) for 24 h. *: significant differences compared to treatment without LPS; #: significant differences between LPS-treated groups (*p*<0.05/Tukeys). B) Treatments with EX-527 or JQ1 had no significant effect on basal or LPS-stimulated ROS generation. A549 cells were treated with EX527 (5 μm), JQ1 (400 nm) and LPS (10 μg mL^−1^) for 24 h. *: significant differences compared to treatment without LPS (*p*<0.05/Tukeys).

**Figure 3:**
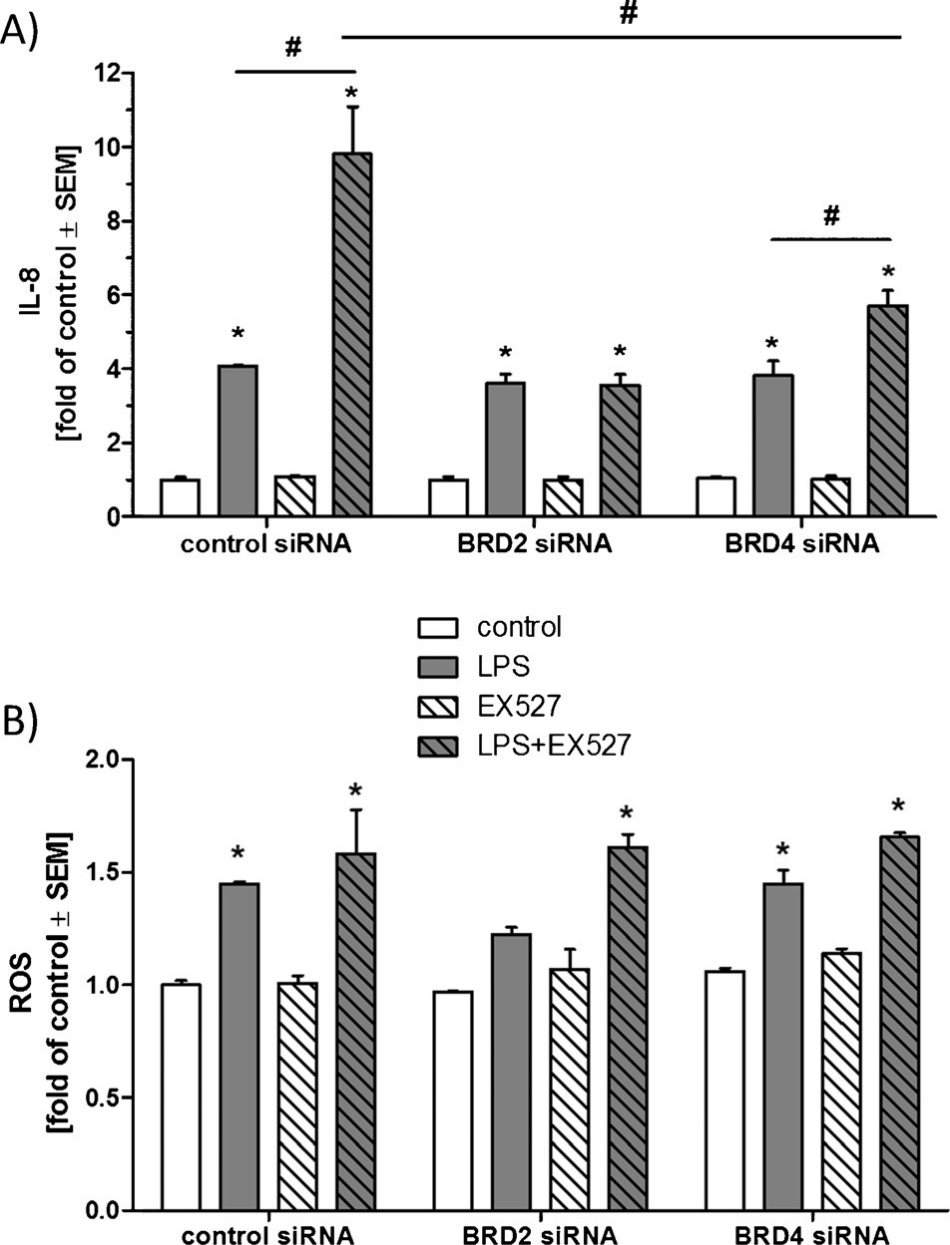
Effects of BRD2 and BRD4 silencing on LPS-induced inflammatory response and ROS generation. A) BRD2 silencing prevented the inflammation-enhancing effect of SIRT1 inhibition. A549 cells were treated with EX527 (5 μm) and LPS (10 μg mL^−1^) for 24 h. *: significant difference compared to treatment without LPS; #: significant difference between LPS-treated groups (*p*<0.05/Tukeys). B) BRD2 silencing reduced LPS-stimulated ROS generation. A549 cells were treated with EX527 (5 μm) and LPS (10 μg mL^−1^) for 24 h. *: significant differences compared to treatment without LPS (*p*<0.05/Tukeys).

Because of the numerous beneficial effects of SIRT1, ways and means of its activation have been eagerly hunted.[3,4] Several selective SIRT1 inhibitors are available for research, but the effectiveness of direct SIRT1 activators is more controversial.[10–15] Also, initial enthusiasm following the original report[16] on the physiological effects of AROS as an SIRT1 activator has faded.[15–18] Therefore, it seems that the most promising way to activate SIRT1 would be by a cellular pathway. In the search for a novel regulation mechanism of SIRT1, we investigated the interplay between BET proteins and SIRT1.

JQ1 upregulates SIRT1 protein levels in several human and mouse cell lines. In line with these results, upregulation of SIRT1 mRNA has been previously detected in microarrays from JQ1-treated lymphocytes.[19] JQ1 also enhanced the deacetylation of tumor suppressor protein p53, a well-established SIRT1 substrate,[20] in human cells. Because JQ1 did not directly modulate SIRT1 activity in a Fluor de Lys in vitro activity assay, the observed increase in p53 deacetylation by JQ1 treatment was most likely a result of increased SIRT1 expression. This suggests that BET protein inhibition not only exposes more acetylated sites for the deacetylase activity of SIRT1, but also upregulates SIRT1 and thus enhances the cellular activity of SIRT1. This will serve as a starting point for developing novel strategies of SIRT1 activation, which would be desirable in many age-related and metabolic diseases.

Human cell line A549 was chosen to investigate the biological responses evoked by JQ1-induced BET inhibition/SIRT1 activation. A549 cells have been extensively used to study alveolar immunotoxicity.[21] They have been used in the study of both chronic inflammation associated with chronic obstructive pulmonary disease, asthma, or cigarette smoke exposure, and acute inflammation associated with influenza, tuberculosis, or pneumonia. They are also often used to model non-small-cell lung cancer, which is the most common form of lung cancer.[22] As chronic inflammation is associated with the pathogenesis of lung cancer,[23] the inflammatory response in A549 cells is important with regard to most the severe lung diseases. Mimicking the intrusion of microbial pathogens by exposure of A549 cells to bacterial LPS has been found to stimulate ROS production, and to evoke an inflammatory response, as demonstrated by NF-kB activation and increased interleukin-8 (IL-8) production.[24] Importantly, activation of SIRT1 by exposure to resveratrol has been found to significantly dampen the inflammatory responses and reverse the effects of LPS in A549 cells, whereas inhibition of SIRT1 was found to result in opposite effects.[25] Therefore, we used IL-8 and ROS production as parameters reflecting the function of epithelial defense mechanisms during the JQ1 treatment.

SIRT1 is an effective inhibitor of inflammatory signaling.[24,27] In contrast, BRD2 and BRD4 are essential for inflammation, and JQ1 treatment has been found to dampen the inflammatory response.[28,29] We found that SIRT1 inhibition enhanced the LPS-induced inflammatory response, whereas BET inhibition had the opposite effect. Furthermore, BET inhibition by JQ1 was able to alleviate the inflammatory response evoked by SIRT1 inhibition. Our results are in agreement with the previously published results about anti-inflammatory effects of SIRT1 activation and BET inhibition. However, our study does not offer final confirmation that the reversal of the pro-inflammatory effect of EX527 by JQ1 occurs primarily through SIRT1 upregulation. Silencing of BRD2 resulted in a complete (and silencing of BRD4 in a partial) reversal of the inflammation-enhancing effect of SIRT1 inhibition. This indicates that both BRD2 and BRD4 are involved in the SIRT1-mediated pathway in A549 cells. The relative importance of these BET proteins on inflammatory signaling has previously been shown to be cell-type specific.[30,31]

In a recent paper JQ1 was found to inhibit ROS production,[32] whereas SIRT1 inhibition by a nonspecific sirtuin inhibitor nicotinamide was previously reported to increase ROS production.[25] In our hands, BET inhibition by JQ1 and SIRT1 inhibition by EX527 had no significant effect on ROS generation. These discrepancies could be explained by the use of different cell types, ROS measurement and exposure times, and by the use of a selective SIRT1 inhibitor in our study. BRD2 silencing was found to reduce the stimulatory effect of LPS on ROS generation. As neither BRD4 silencing nor JQ1 treatment had significant effects on ROS levels, BRD2 silencing is most likely to have its effect on ROS generation through an unknown pathway that does not involve recognition of histone acetylation.

Our results present a novel pathway for SIRT1 upregulation and activation in multiple human and mouse cell types by BET bromodomain inhibitor JQ1 (Figure 4). The inhibitor blocks the interaction of BET proteins with acetylated lysines (Ac in Figure 4), thus allowing the removal of acetylation modifications by SIRT1. This influences the transcription of genes that are induced during inflammatory responses. The inhibition of the binding of BET bromodomains can further influence neighboring histone acetylation modifications. We have also shown that SIRT1 upregulation and bromodomain inhibition have anti-inflammatory effects in a lung disease model, human A549 alveolar epithelial cells, and that both BRD2 and BRD4 bromodomain proteins are involved in these processes. Our study reveals for the first time a functional connection between BET proteins and SIRT1. The BET bromodomains have gained substantial attention for the treatment of human cancers and several inhibitors have already been developed. By combining two classes of epigenetic regulators—BET bromodomains with sirtuins—novel strategies to regulate gene activity are now emerging.

**Figure 4:**
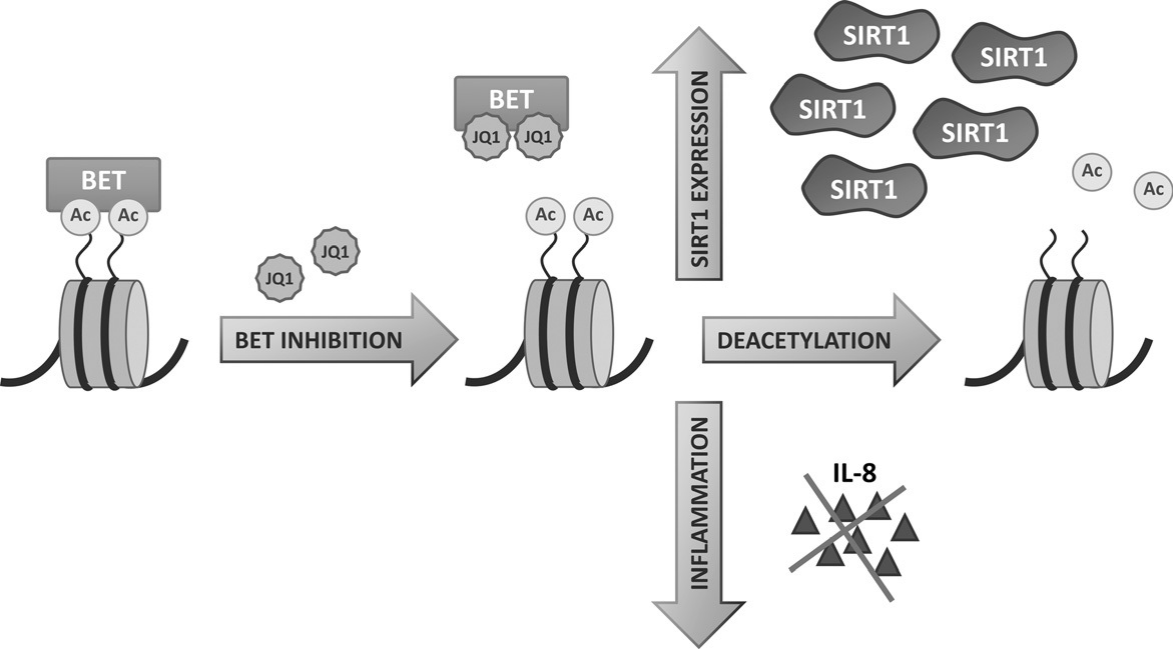
BET inhibition affects SIRT1 expression and function. BET protein inhibition by JQ1 exposes histone acetylation sites (Ac) for the deacetylase activity of SIRT1, upregulates and activates SIRT1 and inhibits inflammation.

## Experimental Section

A549, MCF-7 and HEK293 cells (all from ATCC) were maintained in Dulbeccos modified Eagle medium (DMEM) containing fetal calf serum (10 %), penicillin (100 U mL^−1^) and streptomycin (100 μg mL^−1^, all from Gibco) at +37 °C in a humidified atmosphere of 5 % CO_2_. The mouse N9 microglial cell line was kindly provided by Prof. Paola Ricciardi-Castagnoli (University of Milano–Bicocca, Milan, Italy). Culture conditions were as described earlier.[33] The cells were seeded in 24-well plates (10^5^ cells per well) 24 h before the start of the treatment. Where indicated, etoposide (Sigma–Aldrich) or LPS (L6529; Sigma–Aldrich) was added and incubated for 5 h (etoposide) or 24 h (LPS) before harvesting the cells. For cell-cycle analysis with propidium iodide staining, A549 cells were plated in 6-well plates (0.6×10^6^ cells per well) 6 h before the start of 24 h treatment. Propidium iodide staining and cellular DNA content analysis was as previously described.[7]

Western blot analysis was as previously described.[15] Briefly, cells were lysed in M-PER Mammalian Protein Extraction Reagent (Thermo Scientific) followed by centrifugation (16 000 *g*, 20 min, 4 °C). After separation by SDS-PAGE, the proteins were transferred onto Hybond-ECL nitrocellulose membrane (GE Healthcare) and detected with specific antibodies: rabbit polyclonal SIRT1 C-terminal antibody (ab13749; Abcam, Cambridge, UK); rabbit monoclonal acetyl-p53 (Lys382-Ac) antibody (ab75754; Abcam) and mouse monoclonal α-tubulin antibody (T5168; Sigma–Aldrich). The protein signals were visualised with peroxidase-conjugated secondary antibodies (goat anti-rabbit (ab97051; Abcam) or rabbit anti-mouse (ab97046; Abcam) with chemiluminescent substrate (ECL Plus, GE Healthcare). The images were obtained by digital imaging or by exposure on film. The membranes were reprobed with α-tubulin antibody, and the signal was developed with 3,3′,5,5′-tetramethylbenzidine (T0565, Sigma–Aldrich) as a substrate. The experiments were repeated at least three times, with similar results.

The effect of JQ1 on the enzymatic activity of SIRT1 was measured with Fluor de Lys SIRT1 fluorometric drug discovery activity assay kit (BML-AK555, Enzo Life Sciences, Farmingdale, NY) with substrates BML-KI177 (50 μm, Enzo) and NAD^+^ (500 μm). Fluorescence readings were obtained in a Victor 1420 Multilabel Counter (PerkinElmer) with excitation at 360 nm and emission at 460 nm. The assay was repeated twice.

siRNA gene silencing was done by using Lipofectamine 2000 transfection reagent (LifeTechnologies) according to the manufacturers instructions to transfect A549 cells seeded in 24-well plates (30 000 cells per well) with FlexiTube siRNAs (40 pmol, Qiagen). Four siRNAs were tested for silencing efficiency of each gene (BRD2 and BRD4). AllStars Negative Control siRNA (Qiagen) was used as a negative control. Other treatments were started 24 h post-transfection.

IL-8 determination from cell culture medium was done with an ELISA kit (RAB0319, Sigma–Aldrich). ROS production was assessed by using the fluorescent indicator 2′,7-dichloro-dihydrofluorescein diacetate (H_2_DCFDA) as previously described.[34] Data shown in Figures 2 and 3 are from a single experiment measured in triplicate and analyzed with one-way ANOVA and Tukeys post hoc test in GraphPad Prism. For clarity, only a single level of significance is shown (*p*<0.05). The experiments were repeated at least three times with similar results.

## Supporting Information

As a service to our authors and readers, this journal provides supporting information supplied by the authors. Such materials are peer reviewed and may be re-organized for online delivery, but are not copy-edited or typeset. Technical support issues arising from supporting information (other than missing files) should be addressed to the authors.
