# Supplementary material for: BET Inhibition Upregulates SIRT1 and Alleviates Inflammatory Responses
Source: Chembiochem. 2015 Aug 13;16(14):1997–2001. doi: 10.1002/cbic.201500272 (PMC4600234; doi:10.1002/cbic.201500272)
Supplement: Supplementary file 1 — Supplementary [file cbic0016-1997-sd1.pdf]

CHEMBIOCHEM

Supporting Information

BET Inhibition Upregulates SIRT1 and Alleviates Inflammatory Responses

Tarja Kokkola,*[a, b] Tiina Suuronen,[a] Maija Pesonen,[a] Panagis Filippakopoulos,[c] Antero Salminen,[b] Elina M. Jarho,[a] and Maija Lahtela-Kakkonen[a]

cbic_201500272_sm_miscellaneous_information.pdf

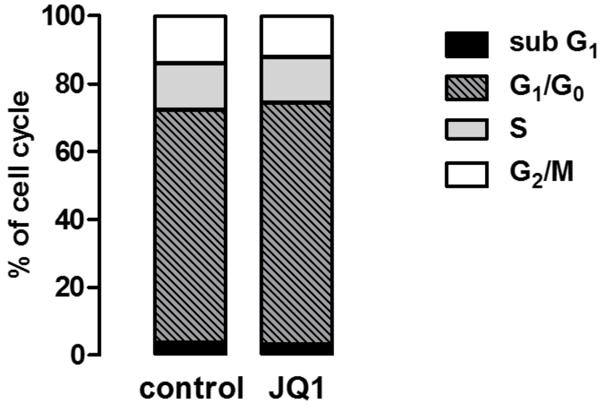

Supplementary Figure 1 | JQ1 did not modulate cell cycle in A549 cells.
The cells were subjected to control treatment (0.5% DMSO) or treatment with 400 nM JQ1 for 24 h. Flow cytometric analysis of DNA content was done after propidium iodide staining. Percentage of cells in each phase of the cell cycle (sub G_1G_1/G_0, S, and G_2/M) is indicated. The results are average of two independent experiments. The errors are omitted for the sake of clarity; the difference between the two experiments was less than 2.5% in all groups.

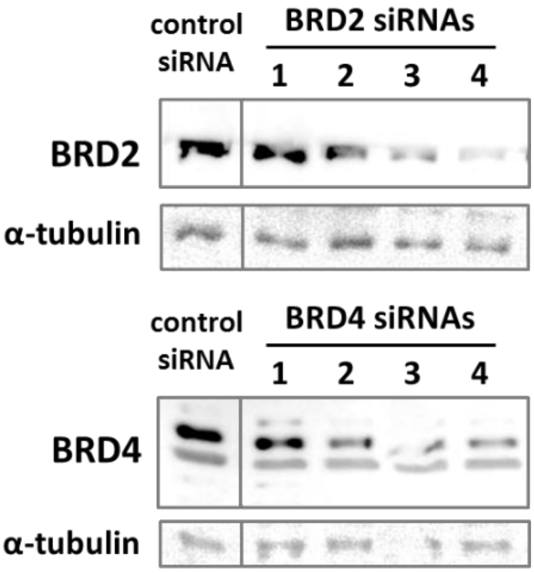

Supplementary Figure 2 | siRNA-mediated gene silencing resulted in diminished protein expression of BRD2 and BRD4.

A set of four predesigned siRNAs were tested for silencing efficiency of each gene. BRD2 siRNA #4 and BRD4 siRNA #4, producing respectively 90% and 75% protein silencing efficiencies, were chosen for further experiments. Cell lysates were analyzed by Western blotting using BRD2 (ab139690, Abcam) and BRD4 (ab75898, Abcam) antibodies and reprobed by α-tubulin antibody (T5168, Sigma). The Western blots are representative of at least three independent experiments.
